# Correspondence regarding the article by Murugan et al. on “Precision net ultrafiltration dosing in continuous kidney replacement therapy: a practical approach”

**DOI:** 10.1186/s40635-023-00585-5

**Published:** 2024-02-20

**Authors:** Rinaldo Bellomo, the Rational Evaluation of Nomenclature and Agreement based on Logic (RENAL) Investigators, Kyle White, Akinori Maeda, Emily See, Lui Forni, Claudio Ronco, Ary Serpa Neto, Thiago Reis, Fernando Zampieri

**Affiliations:** 1 Department of Intensive Care, Austin Hospital, 145 Studley Road, Heidelberg, Melbourne, VIC 3084 Australia; 2 Critical Care, School of Medicine, University of Melbourne, Parkville, VIC Australia; 3 Australian and New Zealand Intensive Care Research Centre, Monash University, Melbourne, VIC Australia; 4 Data Analytics Research and Evaluation, Austin Hospital, Melbourne, Australia; 5 Department of Intensive Care, Royal Melbourne Hospital, Melbourne, Australia; 6 Department of Intensive Care, Princess Alexandra Hospital, Brisbane, Australia; 7 Department of Intensive Care, Austin Hospital, Melbourne, Australia; 8 Department of Nephrology, Royal Melbourne Hospital, Melbourne, Australia; 9 Department of Critical Care, Royal Surrey Hospital Foundation Trust, Guilford, United Kingdom; 10 School of Medicine, Faculty of Health Sciences, University of Surrey, Guilford, United Kingdom; 11 Department of Medicine, Padua University, Padua, Italy; 12 Department of Nephrology, San Bortolo Hospital, Vicenza, Italy; 13 Department of Nephrology, International Renal Research Institute, Vicenza, Italy; 14 Australian and New Zealand Intensive Care Research Centre, Monash University, Melbourne, Australia; 15 Department of Nephrology, Dialysis and Kidney Transplantation, Fenix Nephrology, Sao Paulo, Brazil; 16 Department of Intensive Care Nephrology, Syrian-Lebanese Hospital, Sao Paulo, Brazil; 17 Laboratory if Molecular Pharmacology, Faculty of Health Sciences, University of Brasilia, Brasilia, Brazil; 18 Department of Critical Care, Faculty of Medicine and Dentistry, University of Alberta and Alberta Health Services, Edmonton, Canada

**To the Editor,**

We read the article on precision net ultrafiltration by Murugan et al. [1] with concern. In this article, in addition to the misplaced use of the term continuous kidney replacement therapy instead of continuous renal replacement therapy (CRRT) [2], the main author subverts the very definition of net ultrafiltration (NUF), which he had previously espoused, advocated, and published [3, 4]. Moreover, he destabilises the accepted consensus nomenclature published by the Acute Disease Quality Initiative, which is that NUF is the difference between the effluent removed by the CRRT machine through the process of ultrafiltration and the dialysate and replacement fluid administered via the machine.

In contradiction to their previously determined description and against consensus nomenclature [5], the authors confuse the NUF with the fluid balance (the balance between what is removed by the machine (NUF) and all other fluid losses minus fluid inputs). The article then proceeds (Box 1) to describe an evidence-free process where unnecessary adjustments are made based on incomplete fluid balance. Adjustments that do not take into consideration the complex nature of fluid balance in critically ill patients, such as urinary output (which would occur in polyuric acute kidney injury (AKI), drains (as may be common and substantial after cardiac surgery or abdominal surgery), gastrointestinal losses (as may occur and be substantial in a proportion of critically ill patients), or nutritional input (as a major component of fluid input) are simply wrong.

We acknowledge that people can be free to call a platypus a duck. However, to do so causes unnecessary confusion and impedes the process of reproducible, clear, and consistent clinical research and terminology. As such it needs to be called out as both incorrect and undesirable. We invite the authors to join the rest of the world and adopt the accepted terminology in the future.

## Data Availability

Not applicable.
